# Natural language processing in toxicology: Delineating adverse outcome pathways and guiding the application of new approach methodologies

**DOI:** 10.1016/j.bbiosy.2022.100061

**Published:** 2022-07-28

**Authors:** Marie P.F. Corradi, Alyanne M. de Haan, Bernard Staumont, Aldert H. Piersma, Liesbet Geris, Raymond H.H. Pieters, Cyrille A.M. Krul, Marc A.T. Teunis

**Affiliations:** a Innovative Testing in Life Sciences and Chemistry, University of Applied Sciences Utrecht, Heidelberglaan 7, Utrecht 3584 CS, the Netherlands; b Biomechanics Research Unit, GIGA *In Silico* Medicine, University of Liège, Avenue de l'Hôpital 11, Liège 4000, Belgium; c Centre for Health Protection of the Dutch National Institute for Public Health and the Environment (RIVM), Heidelberglaan 8, Utrecht 3584 CS, the Netherlands

**Keywords:** Natural Language Processing, Adverse Outcome Pathways, New Approach Methodologies, Toxicology, NLP, Natural Language Processing, AOP, Adverse Outcome Pathway, NAM, New Approach Methodology

## Abstract

Adverse Outcome Pathways (AOPs) are conceptual frameworks that tie an initial perturbation (molecular initiating event) to a phenotypic toxicological manifestation (adverse outcome), through a series of steps (key events). They provide therefore a standardized way to map and organize toxicological mechanistic information. As such, AOPs inform on key events underlying toxicity, thus supporting the development of New Approach Methodologies (NAMs), which aim to reduce the use of animal testing for toxicology purposes.

However, the establishment of a novel AOP relies on the gathering of multiple streams of evidence and information, from available literature to knowledge databases. Often, this information is in the form of free text, also called unstructured text, which is not immediately digestible by a computer. This information is thus both tedious and increasingly time-consuming to process manually with the growing volume of data available. The advancement of machine learning provides alternative solutions to this challenge. To extract and organize information from relevant sources, it seems valuable to employ deep learning Natural Language Processing techniques.

We review here some of the recent progress in the NLP field, and show how these techniques have already demonstrated value in the biomedical and toxicology areas. We also propose an approach to efficiently and reliably extract and combine relevant toxicological information from text. This data can be used to map underlying mechanisms that lead to toxicological effects and start building quantitative models, in particular AOPs, ultimately allowing animal-free human-based hazard and risk assessment.

## Highlights

• Natural language processing can support adverse outcome pathways building.
• Natural language processing can support new approach methodologies development.

## 1 Introduction

Guaranteeing the health of humans means that every product coming on the market needs to be thoroughly tested for possible adverse effects on human health. In the past, this has largely been done with the help of animal testing. However, animal testing raises ethical issues and is not a sustainable evaluation method considering the time and financial investments as well as the growing number of substances to evaluate, be it for agricultural, cosmetic, food, medical or energy applications. For that purpose, a new class of evaluation methods, the New Approach Methodologies (NAMs), have emerged. NAMs refer to any non-animal-based method, whether *in vitro* or *in silico* [1].

Adverse Outcome Pathways (AOPs) are a standardized description of toxicological mechanistic information and can form the basis for development of New Approach Methodologies (NAMs). These mechanistic frameworks link molecular triggers to an adverse outcome via a series of steps covering various biological levels, and depend on a wealth of data to provide strong evidence of the relationships between each step [2]. Traditionally, this relies heavily on animal testing. However, several ongoing projects focus on making better use of the knowledge already available in literature, in particular ONTOX [43] and VHP4Safety [45]. Information extraction was previously mostly done manually, while the body of evidence in scientific literature is growing every year. Therefore, new methods are being developed to scan existing literature and extract relevant knowledge from it using machine learning. This paper is focusing on the potential of automated text analysis, better known as Natural Language Processing (NLP), to support and facilitate the process of knowledge extraction for AOP building, leading to the development of NAMs.

The recent advances in NLP and the way they can be applied to support AOPs development will be discussed in the scope of current projects ONTOX and VHP4Safety.

## 2 Adverse outcome pathways in modern toxicology

### 2.1 What are AOPs?

An AOP is a framework to organize existing knowledge pertaining to the mechanisms by which adverse effects are triggered in human organisms [2]. It spans various levels of biological organization, starting with a Molecular Initiating Event (MIE) and ending with one or multiple Adverse Outcome(s) (AO) at the organ, organism or population level. An outcome is considered as AO if it is relevant for hazard and risk assessment. The MIE and AO are connected by a series of molecular, cellular or organ-level events called Key Events (KE). Each pair of KE is connected by a Key Event Relationship (KER) that describes the relationship between the upstream and downstream event and can be causal, mechanistic, inferential or correlation-based. The KER therefore supports and gives quantitative insight for the extrapolation of the downstream KE from the state of the upstream KE [3]. In order to prove the relation between KEs, KERs rely on a wealth of information to feed the weight of evidence (WoE) supporting their existence. In that scope, AOPs can be used to identify critical missing information for hazard and risk assessment. AOP developers can also identify endpoints that can be assessed *in vitro* or *in silico* by a NAM to estimate risk for human, hence contributing to a transition towards animal-free testing [4].

Of course, in reality toxicological mechanisms are more complex. They are neither linear nor one-directional, but rather a network of interactions at the molecular, cellular and organ level, including feedback loops that allow to understand why lower doses of a compound are not toxic despite triggering a MIE. AOPs are therefore intended to be modular and combinable in AOP networks, as in Spinu et al. [5].

AOPs are applicable to multiple species, by focusing on mechanistic relations between KEs rather than apical endpoints in specific species. Indeed, identifying conserved molecular events between species will help regulators extrapolate effects thus reducing the number of animals needed to predict adverse outcomes in humans [6].

Moreover, AOPs are stressor-agnostic: the first element of an AOP is a MIE. This event can be triggered by one or multiple chemicals or other stressors (e.g., radiation, inflammation), but is not defined by a single one, making AOPs much more flexible than previous approaches [3].

### 2.2 AOPs and NAMs

On the chemical level, structure-based models are used to predict chemical activity, such as QSARs (Quantitative Structure-Activity Relationship) [7], but also to evaluate chemical similarity and allow grouping of chemicals, such as RA (Read-Across) [8]. RASAR (Read-Across Structure-Activity Relationship), a combination of the previous two approaches, has also showed promising results. RASAR uses chemical hazard properties as well as structural properties to predict hazard [9]. These methods can be combined with AOPs to allow for more efficient screening of compounds by determining which chemicals are likely to trigger the MIE based on their structural properties. They can therefore help rule entire groups of chemicals in or out for testing, without the need for additional data. By drastically decreasing the amount of chemicals to test, these approaches support the reduction of required animal testing and therefore fall under the denomination of NAMs.

### 2.3 Current approaches building AOPs

The construction of an AOP relies heavily on the gathering and reviewing of extensive data and literature, in order to support confidence and adoption [10]. The OECD [11] has suggested the adoption of tailored Bradford-Hill (BH) considerations to assess the confidence in an AOP. The BH criteria were originally designed to support evaluation of causality of relationships for epidemiological evidence and were adapted to fit the AOP framework. In Becker et al. [12], these criteria are referred to as:

1 Biological plausibility of KERs: what is the level of understanding and acceptance of the biology behind a given KER?
2 Empirical support for KERs: dose-response, temporality, and incidence
3 Essentiality of (KEs): blocking an upstream KE results in the downstream KE not occurring. This can also be proved reversibly, in other words removing the block on the upstream KE leads to the downstream KE happening again.

These criteria are demonstrated in the article by Horvat et al. [13], describing the AOP leading to liver fibrosis from protein alkylation. Horvat et al. [13] followed a top-down approach, selecting an adverse outcome of interest and tracing it back to the key events and MIE(s) of interest. Though AOPs are stressor-agnostic, the authors have used chemicals clinically known to trigger the AO of interest, namely carbon tetrachloride and allyl alcohol, in order to understand the mechanistic process behind the biological response. An overview of all the evidence supporting each KE or KER is also provided, ranging between two and nine supporting scientific articles for each item, totaling close to 180 references for this paper. The authors most likely first had to screen existing literature to select relevant articles. A PubMed search using the keywords liver fibrosis AND (carbon tetrachloride OR allyl alcohol), and limiting the results to before 2017, yielded more than 3500 results https://pubmed.ncbi.nlm.nih.gov/[44]. Reviewing so many papers manually was evidently very cumbersome.

It is clear that AOP building depends on the availability and organization of data. The amount and quality of data used is actually critical to drive confidence and endorsement from peers as well as support usage [14]. Because AOPs are based on manually constructed relationships, WoE determinations are pivotal for their acceptance and usefulness. The BH criteria adapted for application in the AOP framework are a good starting point when considering (semi-)automated construction of AOPs and any computational approach should be built in concordance. Kleinstreuer et al. [15] have already suggested the potential of data science to help provide suggestions of AOP associations from scientific literature [15]. Here we propose to use Natural Language Processing (NLP) to extract AOP-relevant relationships, in concordance with the BH criteria, as one way of moving beyond manual construction of AOPs and into computer-aided extraction of existing knowledge from text.

## 3 Advances in Natural Language Processing

### 3.1 Natural Language Processing techniques and evolution

Machine learning refers to the ability of a computer to learn from data, rather than being explicitly programmed to accomplish a given task [16]. This is often used to mimic human behavior: for example, recognizing a specific person on a picture, understanding a piece of text or determining a causal relationship between two events [17]. Harvesting computing power can multiply scientific discoveries by analyzing large amounts of data and finding previously unknown relationships or knowledge. Machine learning starts with data as an input, and learns a mathematical model on this data. As it operates on mathematical transformations, any input to a machine learning model is numerical.

NLP is a machine learning field concerned with automatically analyzing human language. It has broad application, from characterizing sentiments and detecting hate speech in social media [18,19] to automatic machine translation from one human language to another [20]. When building AOPs, NLP could be used to programmatically retrieve scientific articles, fragment them into paragraphs of interest and automatically mine the relevant relationships between MIEs, KEs and AOs. This information then needs to be stored in a machine-readable way. The AOP developer's role could then focus more on quality control and critical evaluation of the information extracted. In this review paper, we are particularly interested in the progress of these NLP techniques pertinent to extracting information from written text, and specifically the progress of neural networks.

**Fig. 1:**
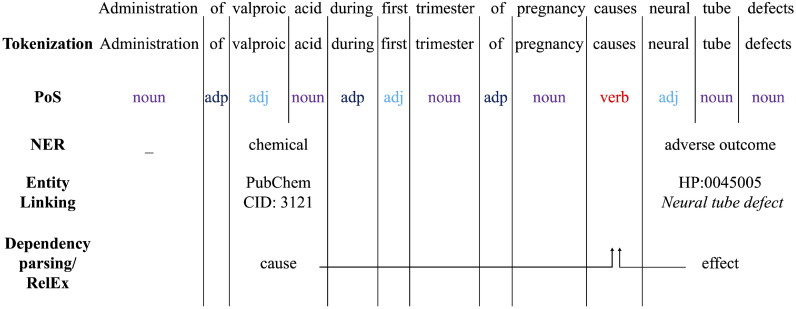
Common NLP tasks, applied to a biological example. PoS: Part-of-Speech tagging, NER: Named Entity Recognition, RelEx: Relation extraction, adj: adjective, adp: adposition, CID: chemical ID, HP: Human Phenotype Ontology term.

NLP can be broken down into several tasks following each other. First, the text of interest is broken into words according to a set of rules depending on the language (grammar, punctuation, etc.) as well as the topic discussed (abbreviations). This first step is called tokenization [21]. Secondly, Part-of-Speech (PoS) tagging maps each word of a sentence to its grammatical function, e.g., noun, verb, adjective, etc. From there, dependency parsing allows to determine the semantic relationships between words, which can potentially lead to discovering relationships between them. So-called “higher level” tasks also build on these preliminary steps [22]. We show an example of some of these tasks applied to a sentence in a biological context in Fig. 1. We see in particular three tasks of interest for information extraction for AOP building:

1 Named Entity Recognition (NER) to recognize concepts such as MIE, KEor AO.
2 Relation Extraction to support KERs: causal relationships feed the WoE.
3 Entity linking assigns a unique identifier to a given (group of) word(s). The advantage is two-fold: disambiguating the word as well as allowing further relations to e.g. existing databases. In Fig. 1, valproic acid is for example linked to its PubChem chemical identifier (CID) [23]. This identifier not only allows the reader to associate the term “valproic acid” with its structure but also to identify synonyms (e.g., “valproate” here) with the same PubChem CID. The information obtained on valproate and valproic acid is then linked as pertaining to the same chemical.

Most NLP tasks can be described as a classification task: assigning a label x to an input text. For example, NER maps a (group of) word(s) (“valproic acid”) to a concept (“chemical”) (Fig. 1). Language processing can further be viewed as a sequence modeling problem: in order to understand a specific part or word of the sentence, we need to be aware of what comes before and after it. For example, the word “expression” can refer among others to a mathematical formula (the expression of the area of a rectangle), the process of translating the information contained in a gene (gene expression) or the emotion conveyed on one's face (facial expression). However, in isolation, it is almost impossible to distinguish which of these meanings the author wants to convey: the context is therefore key to disambiguate. In that regard, some significant progress came from the application of neural networks to language modeling,

A neural network (sometimes also called deep learning) is a type of machine learning architecture built by analogy to the biological concept of a network of neurons. In a neural network, a number of neurons, also called nodes, are densely connected in layers. Each neuron receives a signal from its environment. This environment can be other neurons or input data, and different sources from the environment can have different weights. All the input a given neuron receives is combined via a mathematical transformation. If the combined input is higher than the activation threshold of the neuron, an output is transmitted to the next layer of neurons [24]. The combinatorial possibilities are diverse in terms of number of neurons, number of layers, connection between neurons, etc., and define different architectures [25]. These various architectures can learn complex, non-linear functions mapping an input data (e.g., a sequence of words) to an output of interest (e.g., identification of “expression of gene X” as a KE).

As mentioned previously, the input to any machine learning model is numerical. This means that for text analysis, the first step is to translate words into numbers. One of the major innovations for NLP has been the mapping of words to vectors representing their semantic and contextual similarity [26]. In other terms, words representing concepts that have a similar meaning or are often used together will have similar encodings. These word vectors are usually learned using deep learning on corpora comprising millions of texts such as Common Crawl, a large corpus of several millions of news articles [27].

Word vectors can then be fed into neural networks architecture optimized for sequence modeling, in particular Convolutional Neural Networks (CNN) and Long Short-Term Memory networks (LSTM). CNNs, traditionally used in computer vision, have a particular element of architecture named the convolutional layer. This layer allows to extract features gathering input at different positions of a vector. In other words, this allows to consider “sliding windows” of words across a sentence [28]. On the other hand, LSTMs incorporate a layer allowing to access previous states, in other terms, to remember words coming previously in a sentence. In addition, they include a possibility to forget part of what has already been seen [29]. These advances in neural network architectures improved language models by capturing longer term dependencies and improving sequence modeling of text.

However, these models are still built as a pipeline of models trained and applied one after the other. There is value in sharing information between one task and the next. Information used for PoS tagging, for example, can be equally useful for dependency parsing as they are both relying on grammatical properties. More recently, a new architecture for language models has emerged: the Transformer [30]. Transformer models comprise an attention mechanism, which accounts for the relationships and dependencies between all the words in a sentence, no matter their distance to each other. It assigns differential weights to all words in relation with individual words, allowing to indicate which words are essential for the interpretation of others by giving them a higher weight. The difference with the word vectors concept we presented above is that word vectors are trained on a general corpus. Here, weights are different in each sentence according to the context. This means that the context of a word can be re-used for each task, instead of being learnt multiple times. Transformers have shown very good performance on translation tasks in this founding paper, so it is very likely they will outperform traditional architectures on other NLP tasks as well.

A lot of progress has been made in the NLP field. We will now review how much of this progress has been applied to the field of toxicology.

### 3.2 Current NLP use in the biomedical and toxicology fields

There have been a number of (collective) efforts on the development of language models and applications for the biomedical field in the last ten years [31,32]. Pletscher-Frankild et al. [33] have developed a system to extract gene-disease associations from literature, based on dictionary mapping and co-occurrence. LimTox [34] is a full pipeline and web application designed to retrieve scientific text and extract associations between compounds and toxicological endpoints, focused on hepatotoxicity. However, it is very specifically focused on the extraction of relations between biochemical markers and cytochromes and does not extend to other molecular interactions nor to relations between molecular events and higher level events such as organ or organism-level events. It is also very much rule and pattern-based and does not seem to take full advantage of the promises of NLP for scientific text.

More recently, Minet et al. [35] built ad-hoc gene sets from the mucus hypersecretion AOP developed by Luettich et al. [36]. Publications related to each KE from this AOP were gathered from PubMed, and a list of “seed” genes per KE were extracted manually from this primary corpus. These formed the basis for an extended search which associated these genes with AOP-associated terms such as “smoke” or “tobacco.” Further genes were extracted by NER, and co-occurrence between each pair of genes across the corpus was calculated. After manual curation, the gene set for each KE could be used in gene expression assays to discriminate e.g. samples exposed to cigarette smoke. This work is an example of how NLP can also support the development of new *in vitro* tests to evaluate toxicologically relevant endpoints as well as identify gaps in data.

Jornod et al. [37] have taken NLP one step closer to AOP development by building AOP-helpFinder, a web platform to find existing literature connecting stressors and biological events. This platform is based on the input of existing AOP elements such as MIE/KE/AO. The platform then searches for these elements in the text of existing publications. This search is based on the matching of (a simplified version of the) words comprising the MIE/KE/AO of interest in the text, and calculating a score based on the shortest path between these words. In other words, the closer these terms are in the text, the more likely it is that the tool has correctly identified an event [38]. To the best of our knowledge, this tool identifies events and determines whether they are related by looking if they are co-occurring in text, but is not making use of semantic information to determine causal relationships between events. In addition, this approach seems to mostly be valuable for the extraction of knowledge about already established events, as the user needs to input MIE/KE/AO from AOPWiki or other sources. Therefore, while the tool has established value to uncover new evidence for KERs between existing events [39], the discovery of new events, in particular MIEs or KEs, seems somewhat more restricted.

All these are interesting applications but do not necessarily take advantage of the recent technical progress in NLP. These recent advances have been mostly implemented in the field of pharmacovigilance. Weissenbacher et al. [40] have used deep neural networks, notably LSTMs, to find mentions of medications in tweets. This could be used further to detect those associated with mentions of adverse effects or toxicities. Wang et al. [41] have used a Transformer-based model on case reports from the FDA Adverse Event Reporting System to predict causal relationships between analgesics treatment and liver failure as well as between Tramadol intake and mortality. They report a better performance, and in particular identify more causal factors, than the traditional signal detection methods. This is very valuable, and is getting closer to harvesting the power of NLP for NAMs. However, it is still targeting a restricted part of toxicology by directly linking compounds and Adverse Outcomes without exploring intermediate mechanistic steps.

From these examples, it is clear that some NLP tasks and architectures have been applied in the biomedical and in particular in the toxicology domain for knowledge extraction, and some more advanced techniques for related domains. We think the recent technical progress in NLP can be used further for several aspects of AOPs and NAMs development, and propose our approach in the following section.

## 4 Future of Natural Language Processing in the toxicology field

### 4.1 Opportunities of NLP for toxicology

**Fig. 2:**
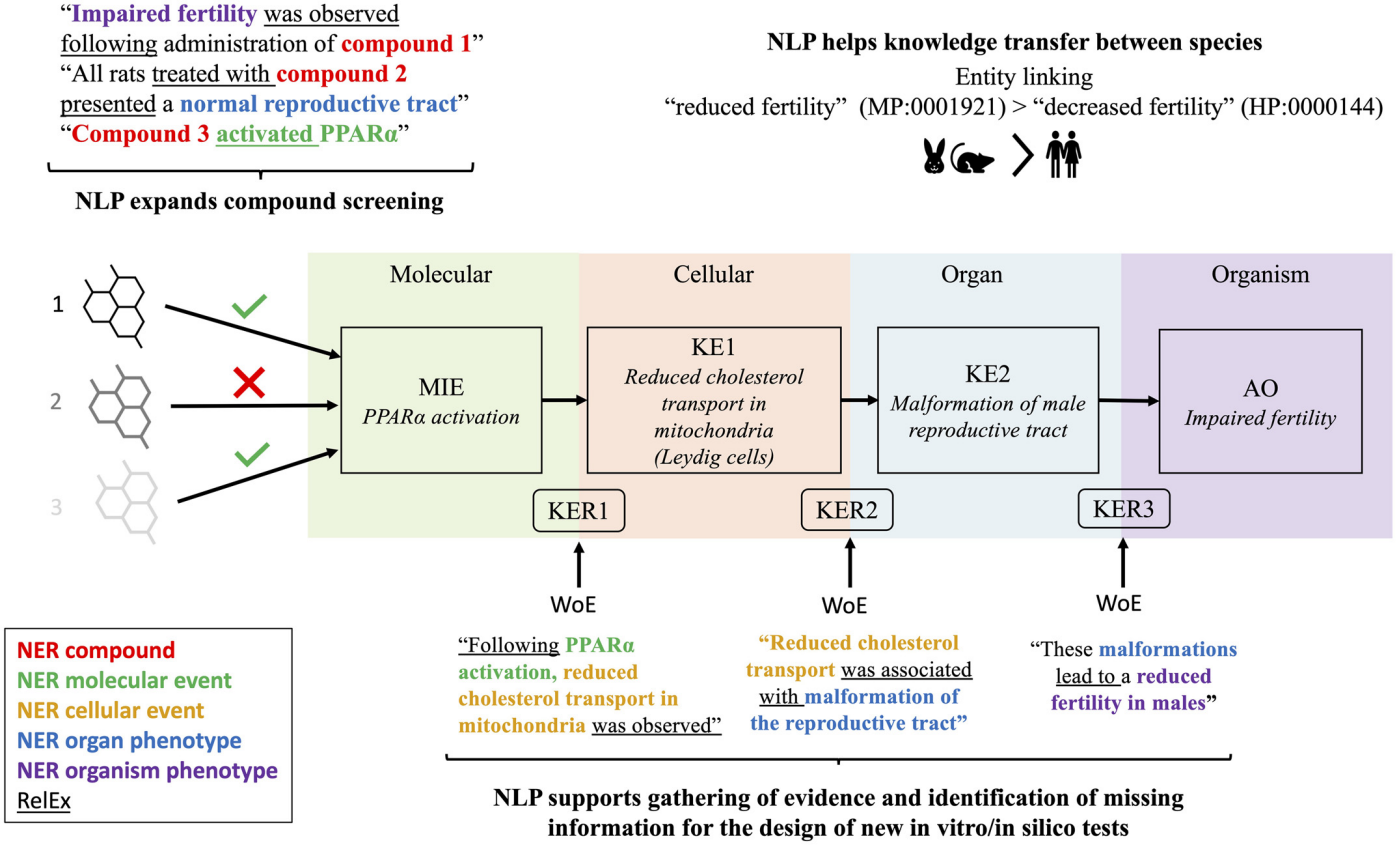
NLP support for AOPs. Here we give an example on how NLP can support the development of AOP with the example of AOP 18 in AOPWiki, PPARalpha activation in utero leading to impaired fertility in males [46]. The example sentences we are giving are meant for illustration and not extracted from any specific article. It should also be noted that we provide one example for each KER, but a lot more would be needed to strengthen and give confidence in the WoE. In addition, by gathering evidence on mechanistic relations between key events that can be present in multiple species, NLP can facilitate the transfer of knowledge between animal models, as well as predict its relevance for human health. MIE: Molecular Initiating Event, KE: Key Event, KER: Key Event Relationship, AO: Adverse Outcome, WoE: Weight of Evidence, NER: Named Entity Recognition, RelEx: Relation Extraction.

We have seen the value that NLP can bring to extract information for toxicological purposes. In Fig. 2, we show the different ways NLP can help NAMs development, with a focus on the AOP framework.

First, connecting chemicals to molecular, cellular, organ or organism-level events can enrich approaches such as RASAR with biological information in addition to the purely physico-chemical parameters already used. The automatic acquisition and addition of that information will then support a more accurate and fine-grained chemical selection. In addition, it can direct toxicological assessment to AOP network areas expected to be triggered by the compound(s), or even to prioritize compounds to be tested, by selecting compounds presenting existing evidence of triggering an AO of interest to test first.

Second, we envision NLP as a support tool for BH criteria. We suggest it can help gather weight of evidence about KERs and support their biological plausibility. For example, highlighting causal relationships between upstream (molecular) key events and downstream (cellular) key events will considerably facilitate the review process by AOP developers. The automation of literature search will also highlight data gaps more efficiently than manual review, which could be filled in by the development of new testing methods (NAMs) as evidenced earlier [35].

Finally, we see NLP as an additional tool to evaluate conservation between species. By extracting biological events and linking them to formally organized concepts such as e.g. ontology terms from the Mammalian Phenotype Ontology [42], we can derive what is conserved in humans from other species for which data exists.

### 4.2 Enacting NLP for toxicology

**Fig. 3:**
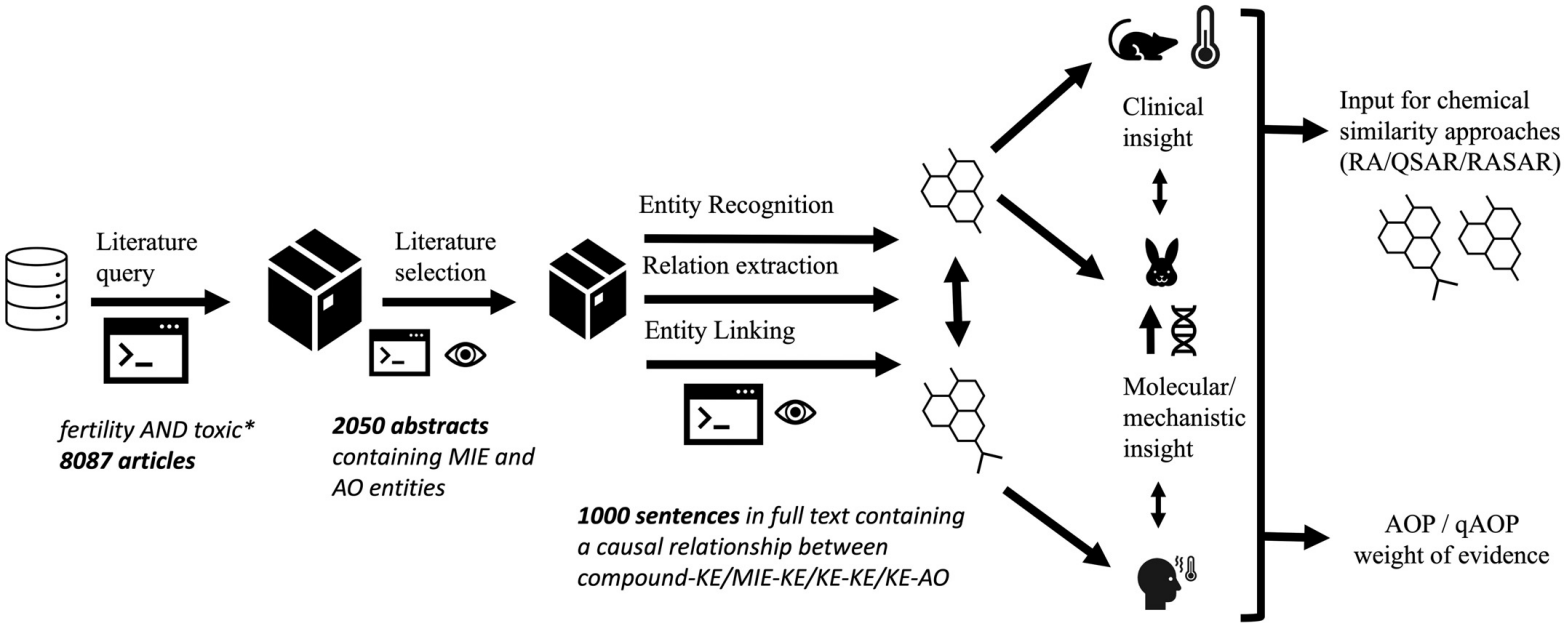
NLP workflow to support gathering of toxicological insights. We give an example of Pubmed query and the number of articles yielded, and how this could be reduced to relevant information (numbers given in the second and third steps of the workflow are theoretical and meant to show how the filtering can be helped by NLP). Example sentences can be found in Fig. 2. This data could then be used for multiple use cases: evaluation of conservation of mechanisms between species, weight of evidence for the establishment of (quantitative) AOPs, or data enrichment for chemical selection process.

We described above how we believe NLP can support the toxicology field. Practically, we suggest a workflow for information extraction, shown in Fig. 3.

The first step is a programmatic query of the relevant literature with well-defined keywords. This can either be a search related to toxic effects of selected chemicals (top-down) or to an AO of interest (bottom-up). As this automatic search will produce an excessive number of results, a curation step is needed. This can be supported by NLP, for example by using NER to recognize biological entities of interest in the text. From then on, the text from selected papers can be processed with the steps we described earlier in Fig. 1. This automatic extraction of information from potentially hundreds or thousands of articles will in our opinion contribute to a more efficient gathering of evidence than manual reviews. This step can and should also comprise some manual inspection from the AOP developer to ensure the models are providing correct information.

All the information is gathered quickly and can be stored immediately in machine-readable format. We envision storing information into graph databases, which would allow easy visualization of relationships and their WoE, for example to support biological plausibility of KERs as exemplified in Fig. 2. It can also be further processed to support NAMs as described in Fig. 2. For example, it can be used as input for similarity approaches to help in chemical screening: chemicals that trigger similar events according to literature could be grouped together.

We believe that this workflow is flexible enough to help the discovery of novel events as well as feed the WoE for KERs within existing AOPs. It can also facilitate combining AOPs into AOP networks.

We see this approach as possibly limited by two factors: the availability of publications in open-science format, as well as the bias for positive results in publications. However, these biases would be similar with a non-automated scheme. We strongly believe that this workflow will be more efficient than a manual approach by accelerating review as well as allowing the evaluation of gaps in currently available knowledge.

## 5 Conclusion

We show here that animal testing-based toxicology can gain from the introduction of new machine learning techniques, in particular NLP, to make better use of the information available in scientific literature. NLP can help describe AOPs, and thereby support the selection of KEs that should be tested in NAMs, driving the selection and novel development of NAMs and paving the way for human-based animal-free chemical hazard and risk assessment.

The workflow we presented here can also be applied to other areas of biomedicine and even Life Sciences in general. We propose that whenever information extraction from an existing body of literature is required, NLP could be applied in this fashion to improve the knowledge gathering process.

## Declaration of Competing Interest

The authors declare that they have no known competing financial interests or personal relationships that could have appeared to influence the work reported in this paper.
